# Influence of *Escherichia coli* chaperone DnaK on protein immunogenicity

**DOI:** 10.1111/imm.12689

**Published:** 2016-12-07

**Authors:** Kirsty D. Ratanji, Jeremy P. Derrick, Ian Kimber, Robin Thorpe, Meenu Wadhwa, Rebecca J. Dearman

**Affiliations:** ^1^ Faculty of Biology, Medicine and HealthSchool of Biological SciencesThe University of ManchesterManchesterUK; ^2^ National Institute for Biological Standards and ControlPotters BarHertfordshireUK

**Keywords:** aggregation, DnaK, host cell impurities, immunogenicity

## Abstract

The production of anti‐drug antibodies can impact significantly upon the safety and efficacy of biotherapeutics. It is known that various factors, including aggregation and the presence of process‐related impurities, can modify and augment the immunogenic potential of proteins. The purpose of the investigations reported here was to characterize in mice the influence of aggregation and host cell protein impurities on the immunogenicity of a humanized single‐chain antibody variable fragment (scFv), and mouse albumin. Host cell protein impurities within an scFv preparation purified from *Escherichia coli* displayed adjuvant‐like activity for responses to the scFv in BALB/c strain mice. The 70 000 MW *E. coli* chaperone protein DnaK was identified as a key contaminant of scFv by mass spectrometric analysis. Preparations of scFv lacking detectable DnaK were spiked with recombinant *E. coli* DnaK to mimic the process‐related impurity. Mice were immunized with monomeric and aggregated preparations, with and without 0·1% DnaK by mass. Aggregation alone enhanced IgM and IgG2a antibody responses, but had no significant effect on total IgG or IgG1 responses. The addition of DnaK further enhanced IgG and IgG2a antibody responses, but only in the presence of aggregated protein. DnaK was shown to be associated with the aggregated scFv by Western blot analysis. Experiments with mouse albumin showed an overall increase in immunogenicity with protein aggregation alone, and the presence of DnaK increased the vigour of the IgG2a antibody response further. Collectively these data reveal that DnaK has the potential to modify and enhance immunogenicity when associated with aggregated protein.

**Abbreviations**
anova: analysis of variance
HCP: host cell protein
HSP: heat‐shock protein
LPS: lipopolysaccharide
NMS: naive mouse serum
OD: optical density
scFv: single‐chain antibody variable fragment
SEC‐MALLS: size exclusion chromatography coupled with multi‐angle laser light scattering
Th1: T helper type 1

## Introduction

Biopharmaceuticals, and in particular therapeutic antibodies, currently make up some of the highest global sales of pharmaceutical products. For example, in 2015 the world's top selling drug was AbbVie's monoclonal antibody Humira (adalimumab).^1^ Biopharmaceuticals, including many antibody fragments that are currently in development,^2^, ^3^ are primarily produced in non‐human host cells, including *Escherichia coli*, rodent cell lines and yeast.^4^ During purification it is well established that small amounts of host cell protein (HCP), including intracellular proteins, will co‐purify with the production biopharmaceutical.^5^ The presence of HCPs in a final drug preparation is referred to as a process‐related impurity,^6^ and such impurities may potentially affect immunogenicity, the production of anti‐drug antibodies, and drug efficacy and/or safety.

Immunodetection‐based methods, such as ELISA, which employ polyclonal antibodies raised against the whole HCP spectrum, are often used for monitoring HCP levels.^7^ Most biologicals submitted for approval to the US Food and Drug Administration contain < 100 parts per million HCPs, as measured by ELISA. It cannot be guaranteed, however, that all HCPs are being measured and with sufficient sensitivity with this method.

The HCPs themselves may be immunogenic but, additionally, they can influence immune responses elicited by exposure to the biotherapeutic, by acting through an adjuvant‐like mechanism.^8^ Such adjuvant activity could lead to the formation of anti‐drug antibodies. HCP contaminants have been shown previously to act as an adjuvant by influencing the immunogenicity of biotherapeutics in the clinic.^9^ For example, a recombinant form of human growth hormone Omnitrope was produced in *E. coli* and an early version resulted in the development of non‐neutralizing anti‐growth hormone antibodies in up to 60% of patients in clinical trials.^10^ The cause of immunogenicity was attributed to HCP contamination and a further purification step was included to reduce the level of HCP impurities. As a result, immunogenicity was significantly reduced.^11^ Hence, bacterial HCPs have the potential to influence the immunogenicity of the biotherapeutic by having adjuvant‐like activity. HCP contamination is also an issue for mammalian cell expression systems.^6^, ^12^, ^13^ However, non‐mammalian HCPs are more likely to pose a risk in patients, compared with their mammalian counterparts.

There is also evidence that HCPs can display adjuvant‐like activity in vaccines. Heat‐shock proteins (HSPs) are molecular chaperones, and bacterial HSPs have been shown to augment adaptive immune responses.^14^ In vaccine development HSPs are now being exploited to improve efficacy.^15^ For example, a novel vaccine strategy for *Neisseria meningitidis* uses a bacterial HSP in a protein antigen–HSP complex that enhances antigen immunogenicity.^16^ HSPs have also found application in cancer immunotherapy, where they are complexed with a tumour antigen, aiding the activation of anti‐tumour immune responses.^17^

The ability of HSPs to enhance immunogenicity emphasizes the need for their identification and control in biotherapeutic formulations. HSPs have been identified in monoclonal antibody preparations with high HCP content purified from Chinese hamster ovary host cells,^18^ and in a study by Schenauer *et al*.,^19^ detectable HCP were identified by using mass spectrometry in purified preparations of an Fc fusion biotherapeutic expressed in *E. coli*. The impact of these HCPs on immunogenicity was not explored; however, within the 20 most abundant *E. coli* cell proteins in the preparation, three HSPs were identified: the 60 000 MW chaperonin GroEL, the chaperone ClpB and the 70 000 MW HSP DnaK. One of the key functions of HSPs is to bind hydrophobic regions on unfolded proteins to prevent aggregation and facilitate protein folding.^20^ As aggregates consist of partially unfolded proteins with exposed hydrophobic regions, it would be anticipated that HSPs are likely to bind with high affinity to these regions. This is of particular relevance for biotherapeutics, where HSPs, which are present as process‐related impurities, might bind to partially unfolded or aggregated proteins. It is widely acknowledged that aggregation itself can contribute to immunogenicity^21^ and it is possible, therefore, that the presence of HSPs could increase further their immunogenic potential.

We have previously shown that aggregates of a humanized single‐chain variable antibody fragment (scFv) caused a T helper type 1 (Th1) ‐skewing of the immune response in BALB/c strain mice.^22^ The purpose of these investigations was to characterize the potential impact of bacterial HCP impurities on the immunogenic activity of protein biotherapeutics using the same system. To this end the scFv and mouse serum albumin were used as test proteins to generate aggregates, and the effect of the addition of the *E. coli* HSP DnaK on immunogenicity was examined.

## Methods

### Single‐chain variable antibody fragment purification

An anti‐c‐met humanized scFv^23^ was cloned into a pET‐22b vector in Shuffle T7 express *E. coli* cells (New England Biolabs, Beverly, MA). Transformants were cultured at 30° to an optical density (OD) of 0·8 at 600 nm, induced with isopropyl *β*‐d‐1‐thiogalactopyranoside and incubated overnight at 16°. Cell pellets were resuspended, sonicated and centrifuged at 28 700 ***g*** for 30 min. The scFv was purified from supernatants using DEAE–Sepharose anion exchange chromatography, followed by Protein A affinity chromatography, through binding the VH region, and size exclusion chromatography.^22^ Protein concentrations were determined by measuring the absorbance at 280 nm using an extinction coefficient of 58 580/M/cm.

For SEC‐MALLS (size exclusion chromatography coupled with multi‐angle laser light scattering) analysis the SEC column outlet was connected to a Dawn Helios MALLS photometer (Wyatt, Santa Barbara, CA) followed by an OptiLab T‐rEX differential refractometer (Wyatt). Data were processed using the wyatt‐qels software (Wyatt, Santa Barbara, CA). Samples were processed by staff at the Biomolecular Analysis Facility at the University of Manchester.

### SDS–PAGE and mass spectrometry

Protein samples were diluted in SDS–PAGE sample buffer (BioRad, Berkley, CA) containing 1% 2‐mercaptoethanol and heated for 3 min at 90°. Samples were resolved on a pre‐cast 10% acrylamide gel (BioRad) and stained using InstantBlue™ Coomassie protein stain (Expedeon, Swavesey, UK). *Escherichia coli* host cell protein levels in scFv preparations were monitored by excising selected bands and analysis by liquid chromatography–mass spectrometry.

Data were processed using the statistical validation software scaffold (version 3.0.04; produced by Proteome Software (Portland, OR)). Samples were processed by staff at the Biomolecular Analysis facility at the University of Manchester.

### Generation of aggregates

*scFv*: Purified monomeric scFv was diluted to 1 mg/ml in Dulbecco's PBS without Ca^2+^ or Mg^2+^ (Sigma‐Aldrich, St Louis, MO) and heated at 40° for 25 min. Aggregates formed were stable and did not dissociate into monomers when the temperature was subsequently decreased by refrigeration, and after storage at −80°.

*Mouse albumin*: Lyophilized mouse albumin (MP Biomedicals, Santa Ana, CA) was diluted to 5 mg/ml in PBS without Ca^2+^ or Mg^2+^ and stressed by heating at 60° for 24 hr. The solution was then diluted to 1 mg/ml in PBS. Aggregates formed were stable and did not dissociate into monomers following dilution and freeze–thaw after storage at −80°. Protein concentrations of mouse albumin were determined from the absorbance at 280 nm using an extinction coefficient of 46 030/M/cm.

### Aggregate analysis: dynamic light scattering

Measurements of dynamic light scattering were performed with a Malvern Zetasizer Nano ZS ZEN3600 (Malvern, Herrenberg, Germany), equipped with a 633‐nm laser. Each sample (70 μl) was measured in a Suprasil^®^ quartz cuvette (Hellma GmbH, Muellheim, Germany) with a path length of 3 mm and 200–2500 nm spectral range. Monomeric and stressed samples at 1 mg/ml were measured at 25° to determine the volume‐based average protein particle diameter in solution.

### Aggregate analysis: circular dichroism

Far‐UV circular dichroism was used to study the secondary structure of the protein before and after heat stress (see Supplementary material, Fig. S1). The measurements were performed with a Jasco J‐815 circular dichroism spectrometer in combination with a Jasco PTC‐423S temperature controller (Jasco International, Tokyo, Japan) at 25°. The samples were measured in quartz cuvettes (Hellma GmbH) with a path length of 1 mm. Circular dichroism spectra were collected in a continuous scanning mode from 190 to 260 nm. The measurements were performed at a scanning speed of 50 nm/min, a response time of 2 seconds, a bandwidth of 1 nm, a sensitivity of 100 m, steps of 0·5 nm, and an accumulation of six scans. Data were analysed using the spectra analysis software dichroweb (University of London, London, UK) and reference data set SMP180.^24^ Data were converted to mean residue ellipticity by the software using a mean amino acid residue mass of 113.

### Animal experiments

Female BALB/c strain mice were used for these experiments (Envigo, Bicester, UK). Mice were housed on sterilized wood bedding with materials provided for environmental enrichment. Food (Beekay Rat and Mouse Diet No.1 pellets; B&K Universal, Hull, UK) and water were available *ad libitum*. The ambient temperature was maintained at 21 ± 2° and relative humidity was 55 ± 10% with a 12 hr light/dark cycle. All procedures were carried out in accordance with the Animals (Scientific Procedures) Act 1986, and approved by Home Office licence. Mice were immunized by intraperitoneal injection with 250 μl of 1 mg/ml protein (monomeric or aggregate) in PBS on days 0 and 7 and exsanguinated on day 14. In some experiments mice received an additional immunization on day 14 and were killed on day 21. In some experiments protein preparations were ‘spiked’ with recombinant *E. coli* DnaK (Enzo Life Sciences, Farmingdale, NY) at a 1 in 1000 dilution (0·1% by mass) relative to the immunizing protein. DnaK was added before and/or after the aggregation treatment. Individual and pooled serum samples were stored at −80° until analysis.

### Antibody ELISA

Plastic Maxisorb^®^ plates (Nunc, Copenhagen, Denmark) were coated with 10 μg/ml scFv or 50 μg/ml mouse albumin in PBS overnight at 4°. The scFv‐coated plates were blocked with 2% (weight/volume) BSA/PBS (Sigma Aldrich) at 37° for 30 min. Mouse albumin‐coated plates were blocked with 5% (weight/volume) skimmed milk proteins in 0·05% (volume/volume) Tween‐20 in PBS for 1 hr at room temperature. Doubling dilutions of serum samples were added (starting dilution 1 in 16, 1 in 32 or 1 in 64) in 1% BSA/PBS for scFv experiments, or 2% skimmed milk proteins in 0·02% Tween‐20 in PBS for mouse albumin experiments and incubated for 3 hr at 4°. Negative control naive mouse serum (NMS) samples were analysed concurrently. Plates were incubated for 2 hr at 4° with horseradish peroxidase‐labelled sheep anti‐mouse IgG diluted 1 : 4000, sheep anti‐mouse IgG1 diluted 1 : 2000, sheep anti‐mouse IgG2a diluted 1 : 1000 (all Serotec, Kidlingtion, Oxfordshire, UK) or goat anti‐mouse IgM diluted 1 : 6000 (Invitrogen, Paisley, UK) diluted in 1% BSA/PBS for scFv experiments, or 2% skimmed milk proteins in 0·02% Tween‐20 in PBS for mouse albumin experiments. Plates were washed between incubations with 0·05% Tween‐20 in PBS. Plates were incubated with substrate *o*‐phenylenediamine and urea hydrogen peroxide for 15 min and reactions were stopped with 0·5 m citric acid.^22^ Absorbance was read at 450 nm using an automated reader (ELx800; BioTek Instruments, Inc., Winooski, VT), using gen 5 1.10 software (BioTek Instruments). Data are displayed as OD_450_ values ± SEM.

### Anti‐DnaK Western blot

For Western blot analysis, protein samples were subjected to SDS–PAGE on 10% acrylamide gels (BioRad) and proteins were transferred onto a nitrocellulose membrane. *Escherichia coli* DnaK was detected using mouse anti‐DnaK IgG1 antibody (Enzo Life Sciences) diluted 1 : 10 000. Blots were incubated with a horseradish peroxidase‐labelled sheep anti‐mouse IgG antibody (Serotec) diluted 1 : 4000 and proteins were visualized using enhanced chemiluminescence reagents (Thermo Scientific, Waltham, MA).

### Statistical analyses

Statistical analyses were performed using the software graphpad prism 6 (GraphPad, San Diego, CA). Analysis of variance (anova) was used to determine statistical significance of differences between more than two groups. Experiments were analysed by non‐parametric one‐way anova followed by the Tukey *post hoc* test, or Student's t‐test (*P < 0·05, **P < 0·01, ***P < 0·001).

## Results

**Figure 1:**
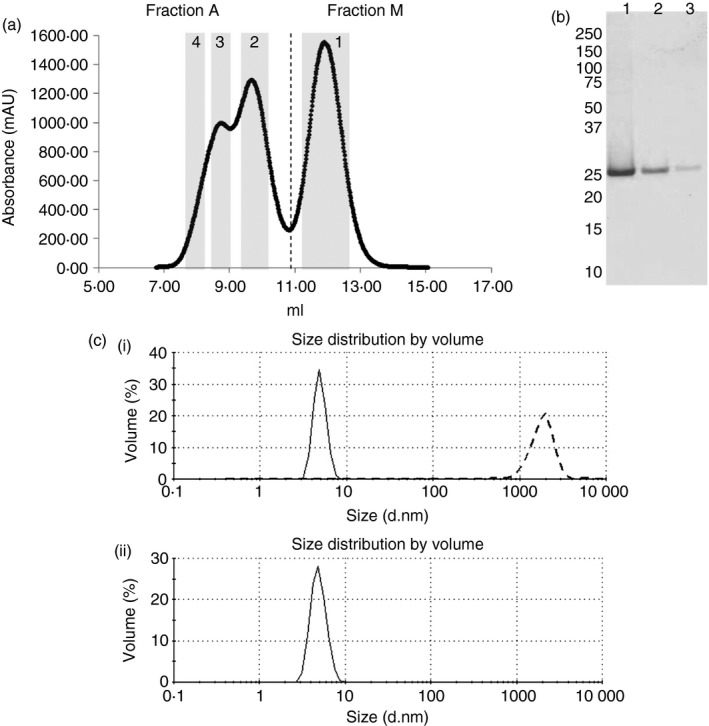
Single‐chain antibody variable fragment (scFv) purification and aggregation. (a) Pooled citrate elution fractions from Protein A affinity chromatography of scFv were fractionated by SEC using a 24‐ml SD75 column, at a flow rate of 0·5 ml/min, and using PBS (pH 7) as a column buffer. The absorbance at 280 nm is presented against the elution volume for a single representative run. SEC‐MALLS analyses were carried out on collected scFv fractions; approximate molecular masses of the fraction shown in grey boxes are: (1) 26 000 (2) 52 000 (3) 80 000 (4) 104 000 (b) scFv fractions were separated by SDS–PAGE and stained using Coomassie Blue. Samples in lanes 1–3 were: Lane 1, Protein A citrate elution before SEC fractionation; Lane 2, Fraction M after SEC fractionation; Lane 3, Fraction A after SEC fractionation. (c) (i) The dynamic light scattering (DLS) profiles of 1 mg/ml Fraction A (oligomeric) scFv in PBS at pH 7 before (solid line) and after (dashed line) heat treatment for 25 min at 40° (ii) The DLS profile of 1 mg/ml Fraction M (monomer).

Our previous work showed that a humanized scFv fragment, which was purified from an *E. coli* expression system, was an ideal test protein to study the effects of aggregation on the immune response.^22^ In the course of investigating protein purification, we analysed the eluted fractions from Protein A affinity chromatography by SEC. The fractions from SEC were subjected to SEC‐MALLS analysis to determine the mass in each peak. The molecular mass estimate of Fraction M was approximately 26 000, and Fraction A was separated into three peaks on SEC‐MALLS, which were approximately: 52 000, 80 000 and 104 000 (Fig. 1a). The higher molecular mass fractions eluted at positions consistent with the formation of dimers, trimers and tetramers of the scFv; these were pooled and designated as Fraction A. The lowest molecular mass fraction from the SEC column was identified as a monomer by SEC‐MALLS, and designated Fraction M. Fractions A and M were run on denaturing SDS–PAGE and stained with Coomassie blue. Protein bands from each fraction had an identical molecular mass, and only one band was observed in each case (Fig. 1b). Incubation at 40° for 25 min of both Fractions A and M resulted in the formation of aggregate populations that were apparently identical by dynamic light scattering, with 2 μm (±1 μm) mean diameter (Fig. 1c) compared with a mean diameter of 7 nm for the monomeric untreated fraction.

### Influence of aggregation on immunogenicity: presence of HCP

**Figure 2:**
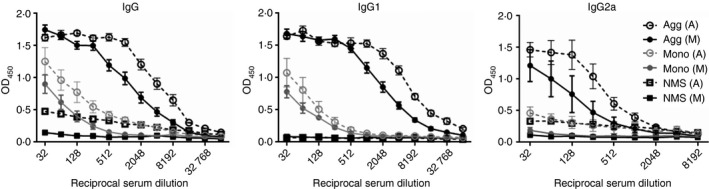
A comparison of single‐chain antibody variable fragment (scFv) Fraction M and Fraction A aggregate immunogenicity. Fraction A scFv was subjected to heat treatment for 25 min at 40°. Mice were immunized by intraperitoneal injection with 250 μl of 1 mg/ml scFv monomer, derived from Fraction Mor heat‐aggregated scFv, derived from Fraction A on days 0 and 7 and serum isolated on day 14 (*n* = 5 per group). Doubling dilutions of serum samples from monomer (Mono) and aggregate (Agg) immunized animals and negative control naive serum samples (NMS) were analysed against both monomeric (M) and aggregated (A) scFv substrates by ELISA for IgG, IgG1 and IgG2a anti‐scFv antibody content. Data are displayed as OD_450_ nm (±SEM) for each reciprocal serum dilution (32–65 536 for IgG and IgG1 antibodies; 32–8192 for IgG2a antibodies). Statistical significance of differences in antibody binding between all sera groups against each substrate was calculated using a one‐way analysis of variance applied to the area under the curve. IgG, IgG1 and IgG2a antibody expression in sera from aggregate‐immunized mice was significantly higher compared with monomer‐immunized mice, against both monomer and aggregate substrates ***P < 0·001.

To investigate the impact of protein aggregation upon immunogenicity, scFv monomer (Fraction M) and aggregate (derived from Fraction A) preparations were administered via intraperitoneal injection to BALB/c strain mice on days 0 and 7, (*n* = 5 per group) and sera were isolated on day 14. The presence of anti‐scFv IgG, IgG1 and IgG2a antibody in serum samples was analysed by ELISA (Fig. 2). Antibody binding was analysed against both monomer (Fraction M) and aggregated (Fraction A heat‐treated) protein substrates and compared with background activity detected in NMS as a negative control.

Analysis of the serum dilution curves revealed that both forms of scFv induced detectable IgG and IgG1 antibody production compared with NMS samples, although only the aggregate provoked significant levels of IgG2a antibody. Indeed, for serum raised against the aggregated material, significantly higher binding was observed against both substrates compared with sera from monomer immunized mice, for total IgG and both subclasses (***P < 0·001). Additionally, higher antibody binding against the aggregate substrate compared with monomer was observed for all serum samples, regardless of the immunization material, with a shift to the right of all titration curves to a broadly similar extent.

**Figure 3:**
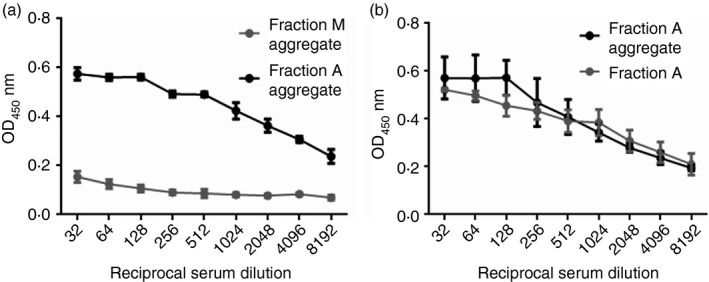
Naive mouse sera (NMS) IgG antibody binding to single‐chain antibody variable fragment (scFv) substrates. Plates were coated with protein substrates at 10 μg/ml. Binding of IgG in naive mouse sera to substrates was measured (*n* = 3 independent analyses of different samples of pooled NMS ± SEM). Doubling dilutions of serum samples (starting 1/32) from naive mice were prepared for incubation in the ELISA. Data are displayed as OD_450_ (±SEM) for each reciprocal serum dilution. Statistical significance of differences in IgG binding between substrates was calculated using a Student's *t*‐test on the area under the curve. (a) IgG binding against heat‐treated fraction A or fraction M. (b) IgG binding against heat‐treated and native fraction A. *P*‐values are cited in the results text where statistically significant differences were achieved.

On monomer‐coated plates, low background OD_450_ readings of < 0·1 were recorded for each isotype in the absence of serum (reagent blank samples) or with NMS. However, there were relatively high levels of binding of NMS against the aggregate substrate for IgG and IgG2a, but not IgG1, detection antibodies, with OD_450_ readings of up to 0·5 recorded. Further ELISAs were carried out to assess whether the high background for NMS samples was due to aggregation *per se* (and hence exposure of neoepitopes following heat treatment) or whether it was due to a contaminant within the high molecular mass Fraction A. IgG antibody present in NMS against native oligomeric scFv from fraction A and the heat‐treated protein was assessed (Fig. 3). Fraction M (monomer) was also aggregated to ~2 μm using heat treatment, and antibody binding was analysed. IgG levels in naive sera directed against aggregated Fraction A were significantly higher than against aggregated Fraction M (***P < 0·001; Fig. 3a). Furthermore, antibody binding against aggregated and non‐aggregated Fraction A was virtually identical. The effect of lipopolysaccharide (LPS) on antibody binding was also investigated (data not shown), and no change in antibody binding was detected when exogenous LPS at 0·025 mg/ml was added to the 0·01 mg/ml monomeric substrate in the ELISA. These results indicated the presence of contaminant(s) in Fraction A that were absent from the monomeric Fraction M, to which there is naturally occurring antibody in naive mice. Mass spectrometric analysis was therefore carried out on Fractions A and M to identify any *E. coli* proteins present.

A number of *E. coli* proteins were identified in Fraction A that were absent from Fraction M, of which the HSPs DnaK and GroEL were the most abundant (Table 1). Given the documented effect of HSPs on adjuvanticity,^14^, ^15^ we hypothesized that these contaminants could be responsible for the enhanced IgG responses for Fraction A versus Fraction M aggregates presented in Fig. 2. The HSP protein DnaK was selected as a candidate to test this hypothesis. SDS–PAGE analysis of scFv spiked with recombinant *E. coli*‐derived DnaK demonstrated that a DnaK protein band was not visible below a concentration of 5 μg/ml (data not shown). Since a band from a low abundance contaminant would not be detectable when Fraction A was analysed by SDS–PAGE/Coomassie staining, it was inferred that the DnaK concentration in Fraction A was < 5 μg/ml. A dose level of 1 μg/ml, which provided a ratio by mass of 1 part DnaK to 1000 parts scFv, was therefore used for further experiments.

**Table 1:** Analysis of the content of Fractions M and A by mass spectrometry

*Escherichia coli* proteins identified	Fraction M	Fraction A
Chaperone protein DnaK		9
60 000 MW chaperonin (GroEL)		6
d‐alanyl‐d‐alanine carboxypeptidase		5
Protein TolB	2	
Tail‐specific protease		2

Fraction A and Fraction M were run approximately 1 cm into an SDS–PAGE gel followed by Coomassie Blue staining. Mass spectrometric analysis for protein identification was carried out by protease digestion and identification by mass. The number of unique peptides that have been matched to the identified protein in the sample is shown in Table 1. The greater the number of matches the more certain the identification. One match is a possible identification, two to three matches a probable identification, four or more show strong positive identifications.

**Figure 4:**
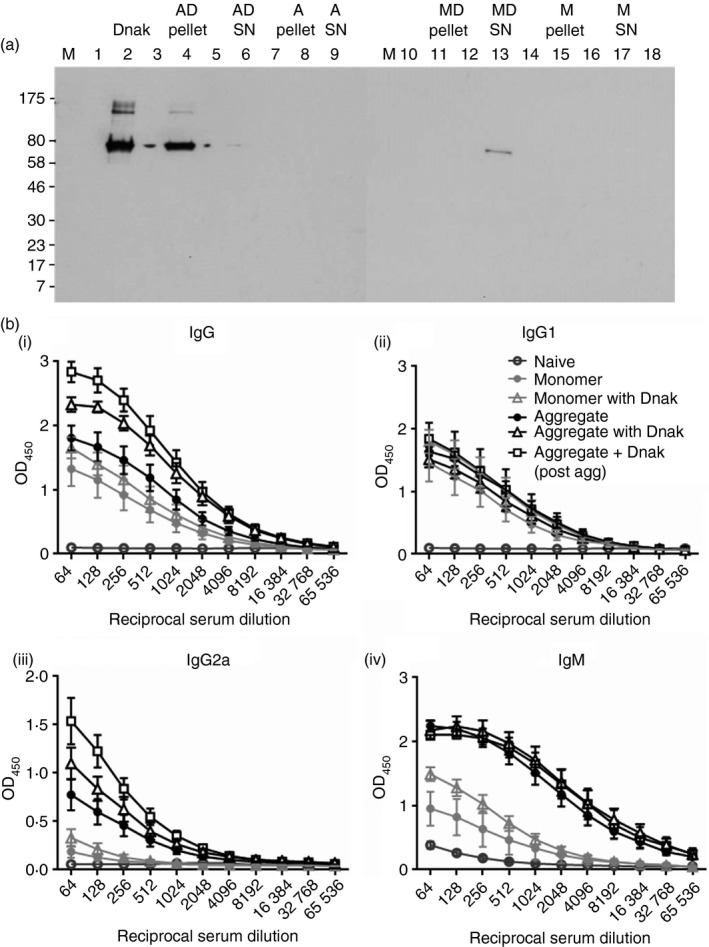
DnaK detection in single‐chain antibody variable fragment (scFv) pellets and supernatants, and antibody responses to scFv monomer and aggregate spiked with DnaK. (a) Western blot of DnaK samples added to monomeric and aggregated scFv. 0·5 μl DnaK (1 mg/ml) was added to 500 μl scFv (1 mg/ml) and either untreated (monomer), or heat treated at 40° for 25 min followed by centrifugation at 12 054 g. Monomeric and aggregated scFv without DnaK were also centrifuged and supernatants and pellets were harvested. For monomeric samples, where pellets were not observed, supernatant was removed after centrifugation and 30 μl PBS was added to the tube to dissolve any unseen pellet. Protein pellets resuspended in 30 μl PBS, 30 μl of supernatants, and 0·5 μg recombinant *Escherichia coli* DnaK were analysed by Western blotting using an anti‐DnaK antibody. A protein marker lane (M) on each gel was used to determine the molecular mass. Lanes labelled 1, 3, 5, 7, 10, 12, 14, 16 and 18 were left blank in case of sample spill‐over. Samples in corresponding lanes are as follows: 2) 0·5 μg DnaK; 4) Aggregate with DnaK (AD) Pellet; 6) AD Supernatant; 8) Aggregate (A) pellet; 9) A supernatant; 11) Monomer with DnaK (MD) pellet; 13) MD supernatant; 15) Monomer (M) pellet; 17) M supernatant. (b) ELISA analysis of antigen‐specific antibody in sera. The scFv at 1 mg/ml in PBS pH 7 was kept alone or spiked with DnaK at 1 μg/ml then subjected to heat treatment for 25 min at 40°; another preparation was spiked with DnaK after heat treatment (post agg). Mice were immunized with 250 μl of these aggregate preparations by intraperitoneal injection on days 0, 7 and 14 and serum was isolated on day 21 (*n* = 6 per group n3 = aggregate + DnaK post agg). Doubling dilutions of serum samples from immunized animals and negative control naive serum samples were analysed against scFv substrate by ELISA for (i) IgG, (ii) IgG1, (iii) IgG2a and (iv) IgM anti‐scFv antibody content. Data are displayed as OD_450_ (±SEM) for each reciprocal serum dilution (64–65 536). Statistical analysis of differences in antibody binding between all immunized groups is summarized in Table 2.

The ability of DnaK to bind selectively to aggregated, as compared with monomeric, scFv was analysed by SDS–PAGE and Western blot (Fig. 4a). After sedimentation of scFv aggregate plus recombinant DnaK, the HSP accumulated in the pellet (lane 4), with very little remaining in the supernatant, with only a very faint band seen in lane 6. Protein concentration was measured in the pellet and supernatant after centrifugation; protein was present in the supernatant at approximately 0·28 mg/ml, whereas the pellet resuspended in 100 μl had a concentration of approximately 3·2 mg/ml, therefore the pellet was enriched with the aggregate, to which DnaK was bound, with some scFv remaining in solution (for the Western blot, the pellet was resuspended in a volume of 30 μl so the whole sample could be run on a gel). Aggregate and monomer without recombinant DnaK contained no detectable DnaK by blot. A control with monomeric scFv plus DnaK that was not subject to aggregation failed to produce a pellet after centrifugation and the DnaK remained in the supernatant (a band was observed in lane 13 but not in lane 11). The detection of DnaK in the monomer supernatant (lane 13) appears fainter than the aggregate pellet (lane 4) because the volume of supernatant was larger than the volume in which the pellet was resuspended.

### Effect of DnaK and aggregation on immunogenicity of scFv

For further immunogenicity experiments the monomeric fraction of scFv from SEC was used to prepare the aggregate and this fraction was confirmed to be free from *E. coli* HCP contamination by mass spectrometry, and by lack of binding of NMS in ELISA and anti‐DnaK antibody in a Western blot.

A sample (1 mg/ml) of monomeric scFv was spiked with DnaK (1 μg/ml; 1000 : 1) and aggregated by heat treatment for 25 min at 40°. Monomer and aggregate protein preparations with and without the addition of DnaK, either before or after aggregation, were administered via intraperitoneal injection (250 μg) to mice on days 0, 7 and 14 and sera were isolated on day 21. IgG, IgG1, IgG2a and IgM antibody binding was analysed in sera from immunized mice against monomeric scFv (Fig. 4b). The results confirmed the baseline immunogenicity of the monomeric scFv observed previously (Fig. 2) with respect to the production of total IgG, IgG1 and IgG2a anti‐scFv antibody. An IgM response was also observed for monomeric scFv (Fig. 4b). Addition of DnaK had no significant impact on the IgG or IgM response to monomer (see Table 2 for statistical analyses of differences). Aggregation was without effect on IgG1 anti‐scFv antibody production, but significantly enhanced IgG2a antibody production (***P < 0·001). Additionally, aggregation alone significantly enhanced the anti‐scFv IgM response (***P < 0·001). This effect of aggregation on IgM antibody levels has previously been reported.^22^ The addition of DnaK enhanced the IgG2a anti‐scFv response in aggregated preparations (**P < 0·01), but was without effect on IgG1 or IgM antibody production. We infer that the increase in total IgG signal was due to an increase in IgG2a, as IgG1 antibody production was not up‐regulated. The impact of DnaK on antibody binding was comparable when DnaK was added before or after heat treatment (total IgG: ***P < 0·001 monomer versus aggregate with DnaK added before or after aggregation).

**Table 2:** Statistical analysis of antibody quantification by serum dilution

Antibody isotype	*P* value	Condition 1 (immunizing material)	Condition 2 (immunizing material)
scFv + DnaK (Fig. 4)			
Total IgG	< 0·001***	Monomer with or without DnaK	Aggregate with DnaK
Total IgG	< 0·01**	Aggregate	Aggregate with DnaK
IgG1	No significant differences between immunized groups		
IgG2a	< 0·001***	Monomer	Aggregate with or without DnaK
IgG2a	< 0·01**	Aggregate	Aggregate with DnaK
IgM	< 0·001***	Monomer with or without DnaK	Aggregate with or without DnaK
Mouse albumin (versus aggregate substrate only; Fig. 7)			
Total IgG	< 0·05*	Monomer	Aggregate
Total IgG	< 0·001***	Monomer with DnaK	Aggregate with DnaK
IgG1	No significant differences between immunized groups		
IgG2a	< 0·001***	Monomer with or without DnaK	Aggregate with or without DnaK
IgG2a	< 0·05*	Aggregate	Aggregate with DnaK
IgM	< 0·05*	Monomer with or without DnaK	Aggregate

For antibody data presented in Figs 4 and 7, statistical significance of differences in antibody binding between all immunized sera groups were calculated using a one‐way analysis of variance applied to the area under the titration curve (*P < 0·05, **P < 0·01, ***P < 0·001). Results are presented where values of *P* < 0·05 were achieved between the two immunization conditions (condition 1 and condition 2).

### Adjuvant‐like effect of DnaK on mouse albumin immunogenicity

**Figure 5:**
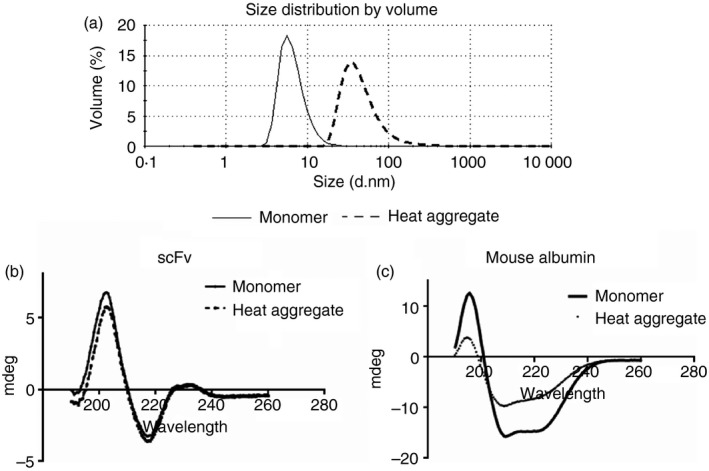
Dynamic light scattering (DLS) and circular dichroism spectra of native and heat‐treated proteins. (a) DLS of mouse albumin. Mouse albumin at 5 mg/ml in PBS pH 7 was heated for 24 hr at 60°, then diluted to 1 mg/ml in PBS. The mean particle diameter was measured by DLS before (i) and after (ii) the heat treatment. A change in mean particle size from 7 to 50 nm was observed following heat treatment. Circular dichroism measurements were recorded using a Jasco J‐815 circular dichroism spectrometer at 25°. (b) scFv was measured at 1 mg/ml in PBS as monomer, or after heat treatment at 40° for 25 min (dashed line). (c) Mouse albumin was measured as monomer at 1 mg/ml in PBS. Mouse albumin was stressed 5 mg/ml in PBS by heating at 60° for 24 hr; the solution was then diluted to 1 mg/ml in PBS before measurement (dotted line).

Human proteins are used in a clinical setting, and trigger a much lower immune response in the absence of additional aggravating factors, such as aggregation. We therefore set out to investigate whether the adjuvant‐like effect of DnaK could be reproduced with a murine protein. Mouse albumin was selected as a well‐characterized, commercially available ‘self’ protein. The adjuvant effect of DnaK on the response to mouse albumin, in monomeric and aggregated forms, was then investigated in a similar fashion to scFv. A number of strategies were attempted to induce aggregation, including a range of temperature treatments, incubation times, stir stress, and the use of various buffers. Mouse albumin required harsher conditions than the scFv to reproducibly form aggregates: we identified heat treatment at 60° for 24 hr as the optimum condition, generating a mean particle size of ~50 nm (Fig. 5a). This was somewhat smaller than the scFv aggregates, which had a mean particle size of 2 μm. Given the relatively high temperature required, the extent of unfolding of the protein was examined by circular dichroism analysis. Monomer and heat aggregates of both scFv and mouse albumin were analysed; whereas the scFv demonstrated little unfolding following aggregation, an overall reduction of about 50% in secondary structure content was observed after heat treatment of the mouse albumin (Fig. 5b,c). These data further highlight the point that the aggregation treatments for the scFv and mouse albumin are not comparable.

Recombinant *E. coli* DnaK was added into 1 mg/ml heat‐aggregated mouse albumin to a final concentration of 1 μg/ml, and its association with DnaK analysed by Western blot. As the mouse albumin aggregates (~50 nm) were much smaller than the scFv aggregates (~2000 nm), centrifugation at 12 054 g was insufficient to separate the aggregates and ultracentrifugation at 802 000 g was required. However, in this case the concentration of mouse albumin present in the supernatant after centrifugation was approximately 0·45 mg/ml, and 4 mg/ml in the pellet when resuspended in a volume of 100 μl, indicating that only a proportion of the aggregate was fractionated into the pellet (which appeared visibly smaller than the pellet achieved with the scFv aggregate under the same conditions).

**Figure 6:**
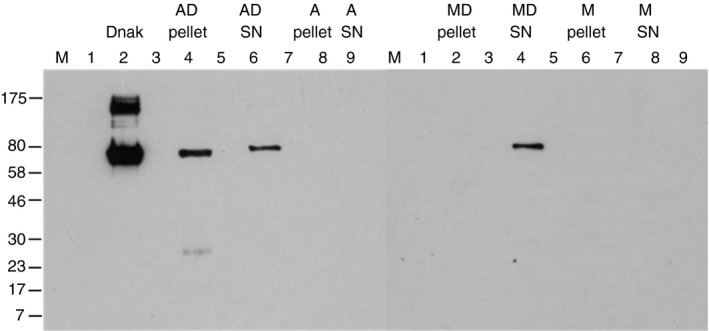
DnaK detection in mouse albumin pellets and supernatants. Mouse albumin was heat treated at 60° for 24 hr at 5 mg/ml, then diluted to 1 mg/ml and kept alone, or 0·5 μl DnaK (1 mg/ml) was added to 500 μl aggregated mouse albumin. Mouse albumin was also diluted to 1 mg/ml and kept alone or with the addition of 1 μg/ml DnaK. Samples were subjected to ultracentrifugation at 802 000 g for 30 min, and supernatants and pellets were harvested. For monomeric samples, where pellets were not observed, supernatant was removed after ultracentrifugation and 30 μl PBS was added to the tube to dissolve any unseen pellet. Protein pellets resuspended in 30 μl PBS, 30 μl of supernatants, and 0·5 μg recombinant *E. coli* DnaK were analysed by Western blotting using an anti‐DnaK antibody. A protein marker lane (M) on each gel was used to determine the molecular mass. Lanes labelled 1, 3, 5, 7, 10, 12, 14, 16 and 18 were left blank in case of sample spill‐over. Samples in corresponding lanes are as follows: 2) 0·5 μg DnaK; 4) Aggregate with DnaK (AD) Pellet; 6) AD Supernatant; 8) Aggregate (A) pellet; 9) A supernatant; 11) Monomer with DnaK (MD) pellet; 13) MD supernatant; 15) Monomer (M) pellet; 17) M supernatant.

Western blot analysis of DnaK content demonstrated that the HSP was present in both the pellet and supernatant in similar amounts, although the pellet fraction appeared slightly larger (Fig. 6, lanes 4 and 6). The distribution of DnaK between supernatant and pellet fractions is therefore consistent with preferential binding of the HSP to albumin aggregate, in a similar fashion to the scFv aggregates in Fig. 4. Aggregate and monomer without DnaK added contained no detectable DnaK by blot, and DnaK did not sediment under these conditions when it was added to the monomer, but remained in the supernatant (lanes 11 and 13).

**Figure 7:**
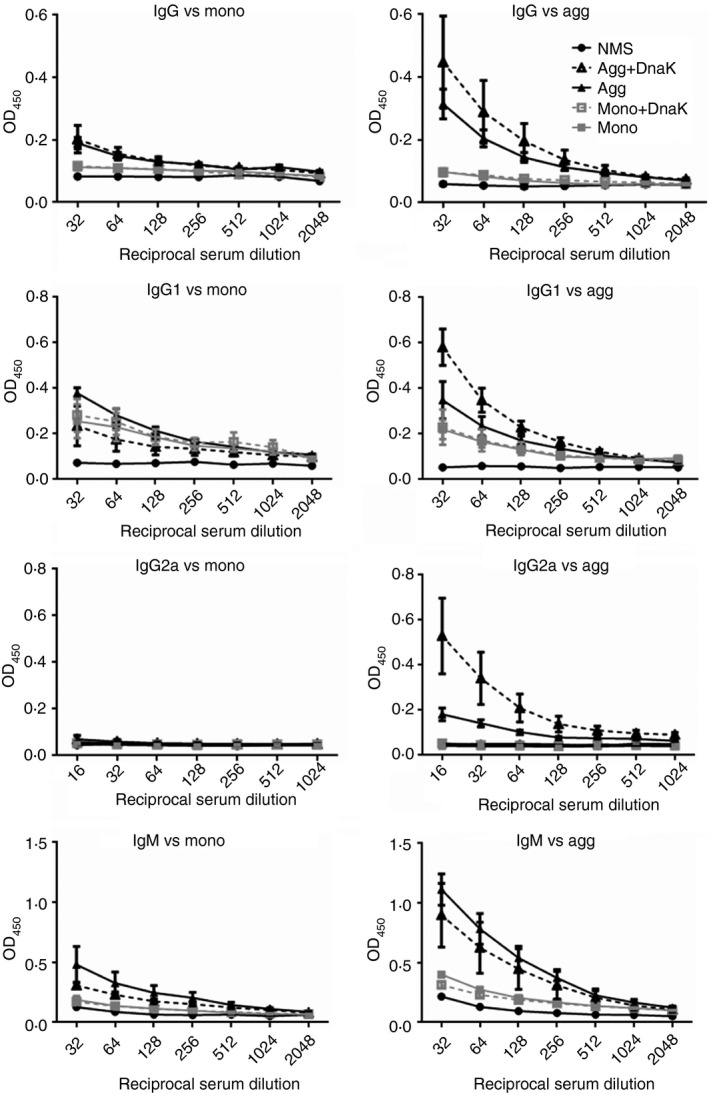
Characterization of immune responses to mouse albumin: comparisons of monomer and heat‐stressed aggregates. Mouse albumin at 5 mg/ml in PBS pH 7 was subjected to heat treatment for 24 hr at 60°, then diluted to 1 mg/ml in PBS. Mice were immunized by intraperitoneal injection with 250 μl of 1 mg/ml monomer or heat‐aggregated mouse albumin, with or without the addition of DnaK at 1 μg/ml on days 0, 7 and 14 and serum isolated on day 21 (*n* = 3 per group). Doubling dilutions of serum samples from single‐chain antibody variable fragment (scFv) monomer (Mono) and aggregate (Agg) immunized animals and negative control naive serum samples were analysed against both scFv substrate proteins [versus monomer (left) and versus aggregated protein (right)] by ELISA for IgG, IgG1, IgG2a and IgM anti‐scFv antibody content. (i) Data are displayed as OD_450_ (±SEM) for each reciprocal serum dilution (1 in 32 to 2048 for IgG, IgG1 and IgM antibodies; 1 in 16 to 1 in 1024 for IgG2a antibodies). Statistical analysis of differences in antibody binding against the aggregated substrate between all immunized groups is summarized in Table 2.

Mice were immunized with mouse albumin monomer or aggregate, with or without the addition of DnaK (at a 1 : 1000 ratio). To determine whether identical epitopes were present on both monomeric and aggregated proteins, parallel plates were coated with monomer or aggregate substrates. IgM, IgG, IgG1 and IgG2a antibody binding was analysed in sera from immunized and naive mice against both substrates (Fig. 7). As expected, IgG, IgG2a and IgM levels in serum from monomer‐immunized animals were identical to NMS, regardless of the presence of DnaK, against both monomeric and aggregated substrates. Low levels of IgG1 antibody were detected, but this was independent of substrate used and was also unaffected by the presence of DnaK. Immunization with the aggregate resulted in a more vigorous immune response for all antibody isotypes, but this was dependent on the substrate used in the ELISA, with the aggregated substrate showing much higher antibody binding. Total IgG, IgG1 and IgG2a anti‐mouse albumin antibody responses were enhanced by the presence of DnaK in the aggregated preparation. The enhanced response with DnaK was most notable for the IgG2a isotype, where a clear significant difference was observed between aggregate and aggregate with DnaK immunized groups (*P < 0·05; Table 2).

## Discussion

Preparations of biotherapeutic proteins inevitably contain HCP impurities. For the purposes of clinical administration, difficulties arise in deciding on an acceptable level of HCP impurities. Such considerations need to be based on evidence that, at particular levels, HCPs may impact on therapeutic efficacy and/or safety. To date the focus has been primarily on the identification and quantification of HCPs, the aim being controlling the levels of these proteins,^25^ and evaluation of their potential role as antigens.^7^ Here we sought to address a somewhat different question regarding the adjuvant properties, rather than the immunogenic potential, of HCPs. For this purpose we chose to consider the potential of HCP impurities to display adjuvant properties and modify and/or enhance the immune response to aggregated and non‐aggregated third‐party proteins.

Initially, we observed that an oligomeric aggregate fraction of a humanized scFv resulted in a more vigorous anti‐scFv response than did the monomer. Further analysis identified the presence of *E. coli* HCPs, including HSPs, in the oligomeric population. It is well established that protein aggregation has the potential to contribute to immunogenicity.^21^ Furthermore, the principal function of HSPs is to mediate the correct folding of proteins inside the cell and therefore, by nature, they bind to partially unfolded proteins.^20^ We also noted that the ability of HSPs to enhance the immunogenicity of a co‐administered antigen is well‐documented, with the successful use of HSPs in vaccine development.^15^ We accordingly selected the *E. coli* HSP DnaK, and studied this individually as a candidate HCP impurity. However, it is possible that the presence of multiple HCPs may have a synergistic effect, and could explain why any adjuvant effect of DnaK alone might not be as strong as the effect of combined HCP impurities. We examined the effects of DnaK on the immunogenicity of aggregated forms of two different proteins. One, an scFv, was selected because we have characterized its immunological responses previously and it displays a relatively vigorous baseline immunogenicity.^22^ We also studied mouse albumin, as an exemplar protein for *in vivo* immunogenicity assessment as it would be treated as ‘self’ by the BALB/c mice.

The requirement of HSP binding to peptides to elicit immunogenicity has been shown previously: for example, HSP 70‐associated peptides derived from cancer tissues elicited tumour‐specific immunity.^26^, ^27^ We therefore examined the ability of DnaK to bind to aggregates; Western blot analysis demonstrated that DnaK co‐sediments with scFv aggregates, and was therefore bound to aggregates rather than remaining in solution. It is proposed that heat treatment of the scFv exposed hydrophobic regions on the protein, leading to aggregation and also enabling binding of the HSPs. Binding studies by Western blot using mouse albumin were also consistent with DnaK binding preferentially to aggregates. Incomplete sedimentation of the mouse albumin aggregates may be due to their small size; however, detection of DnaK in the pellet does show that DnaK was bound to the aggregated protein. It is possible that the higher temperature used for heat treatment of mouse albumin compared with the scFv resulted in a more complete unfolding and denaturation of the protein structure. A circular dichroism spectrum of the aggregated material supports this hypothesis and indicates a more disrupted secondary structure after stress treatment of the mouse albumin.

Antibody analysis of serum samples from mice immunized with scFv monomer or aggregate (free from DnaK, as verified by mass spectrometry analysis), alone or spiked with recombinant *E. coli* DnaK, highlighted differences in the IgG2a response between monomeric and aggregated scFv. This was expected as we have previously shown that subvisible‐sized aggregates of scFv promote a Th1‐skewed response.^22^ It is possible that the Th1 skewing is an effect of differential antigen processing and presentation, and this effect is also consistent with the hypothesis that the repetitive epitopes formed by aggregation can mimic characteristics of microbes and viruses.^28^ The anti‐scFv response was higher still with the addition of DnaK, but in aggregated preparations only, where an increase in anti‐scFv IgG and IgG2a was observed. This suggests an amplification of the Th1‐skewed response with the presence of DnaK, as there was no difference in IgG1 between immunized groups. Importantly, the increased response occurred regardless of whether DnaK was added to the scFv preparation before or after heat treatment, indicating that the presence of DnaK during protein unfolding was not necessary for DnaK binding to the aggregates. This suggests that once the therapeutic has begun to aggregate, endogenous HSPs may also associate with the unfolded protein.

In these experiments a dose of 250 μg scFv was used in mice, and so, for groups immunized with DnaK, each dose provided 0·25 μg of DnaK. Even after sophisticated purification steps, low levels of HCPs may still remain in a final purified biotherapeutic,^29^ although it is important to note that the ratio of DnaK : scFv used in these experiments (1 : 1000) was higher than would be expected in most biotherapeutic preparations used clinically. Accepted limits of HCPs are generally 1–100 parts per million.^29^ However, 1 part per million HCPs could result in 0·1 μg HCP in a 100‐mg dose (biotherapeutics given to patients can reach doses well above 100 mg^30^, ^31^). HSPs, by nature, bind with high affinity to partially unfolded proteins with exposed hydrophobic segments and aggregates^32^, ^33^ and so, even at very low levels will bind preferentially to any aggregates present and therefore have the potential to affect protein immunogenicity.

The scFv experiment was repeated using mouse albumin as the antigen. Mouse albumin was chosen as it is a commercially available murine antigen that could be aggregated within the subvisible size range. It also differs both in size and structure to the scFv, so was ideal to study as an alternative protein. Using a mouse antigen also allows investigations to mimic more closely the situation in humans where patients elicit an immune response to human‐derived biotherapeutics. Antibody analysis of sera from mouse albumin‐immunized mice highlighted increased binding of aggregate immunized mouse sera to the aggregated protein substrate, and little or no binding to the monomeric substrate. This suggests that the heat treatment at 60° required for mouse albumin aggregation resulted in the formation of immunogenic neo‐epitopes that were not recognized by sera from monomer immunized mice. While there was no clear adjuvant effect observed with the addition of DnaK at the level of total IgG, the IgG2a response was higher with the addition of DnaK to the aggregate (*P < 0·05), indicating that the Th1 skewing, which we have also observed with other aggregates, is enhanced when DnaK is present. An increase in IgM was also observed with aggregation of both the scFv and mouse albumin, with no effect when DnaK was present. This was not expected after a 3‐week immunization protocol as IgM production is a primary response, and the IgG analyses demonstrated that class‐switching had occurred. It is possible that the persistence of IgM after three immunizations may be due to immature B‐cell activation caused by antigen repetitiveness on the aggregates.

The adjuvant effect observed with DnaK contamination of scFv and mouse albumin aggregates is consistent with observations made by other investigators. It is well documented that HSPs are capable of exerting an adjuvant‐like effect,^34^ although this is the first demonstration, to our knowledge, that they can amplify responses to protein aggregates. It is difficult, at this stage, to speculate on the immunological mechanisms that might underpin this effect. It is possible that DnaK interacts directly with receptors on immune cells, enhancing uptake of the aggregate. It has been reported that HSPs interact with and activate the immune system via Toll‐like receptors on antigen‐presenting cells.^35^, ^36^ For example, HSP 70 has been shown to stimulate cytokine and chemokine production in dendritic cells,^37^ and it has been speculated that this stimulation is due to HSP 70 interaction with the CD40 receptor, to which mycobacterial and human HSP 70 are known to act as alternative ligands.^38^, ^39^ Other receptors present on antigen‐presenting cells that have been implicated in HSP interaction include CD91, scavenger receptors, c‐type lectin receptors and LOX‐1.^40^ It is also possible that aggregation itself enhances antigen uptake, and the presence of DnaK within the complex somehow enhances processing and presentation once inside the antigen‐presenting cell.

This study only investigated the effect of exogenous bacterial HSP contamination on protein immunogenicity. It is possible that endogenous HSPs in patients may also interact with aggregates once administered. Exogenous HSPs in the biotherapeutic formulation or endogenous HSPs *in vivo* could, in theory, complex with aggregates and so be presented to the host immune system in a more ‘immunogenic’ form. Clinical reports demonstrate that HSP levels are elevated in the plasma of patients with certain illnesses such as dyslipidaemia,^41^ coronary heart disease,^42^ prostate cancer^43^ and neurodegenerative disease.^44^

The data here provide evidence that low levels (1 part per 1000 ) of the HSP DnaK can act as an adjuvant to a co‐administered antigen. Clearly further investigations will be required to establish the degree to which this phenomenon has implications for the clinical use of protein therapeutics. Nevertheless, these results highlight important areas that might form the focus of future work. For example, it will be important to determine whether, and to what extent, other HSPs have a similar potential to exert adjuvant‐like activity.

In conclusion, we report that while aggregation alone can increase the immunogenicity of a mouse protein, and selectively enhance the IgG2a response to a humanized scFv, the addition of DnaK to either protein can enhance further the IgG2a response. Based on these results, it is proposed that the association of certain HSPs with proteins may contribute importantly to the enhanced immunogenicity displayed by aggregated proteins, possibly via promotion of Th1‐type immune responses.

## Disclosures

This work was funded by the Bioprocessing Research Industry Club (BRIC) and the Biotechnology and Biological Sciences Research Council (BBSRC). The authors have no financial or commercial conflicts of interest to disclose.
